# High‐Performance P‐Channel Tin Halide Perovskite Thin Film Transistor Utilizing a 2D–3D Core–Shell Structure

**DOI:** 10.1002/advs.202104993

**Published:** 2021-12-19

**Authors:** Junghwan Kim, Yu‐Shien Shiah, Kihyung Sim, Soshi Iimura, Katsumi Abe, Masatake Tsuji, Masato Sasase, Hideo Hosono

**Affiliations:** ^1^ Materials Research Center for Element Strategy Tokyo Institute of Technology 4259 Nagatsuta Yokohama 226–8503 Japan; ^2^ WPI‐MANA National Institute for Materials Science Tsukuba 305‐0044 Japan; ^3^ Silvaco Japan Co., Ltd. Nakagyo‐ku Kyoto 604–8152 Japan

**Keywords:** complementary metal oxide semiconductors (CMOS) inverter, core–shell structures, Pb‐free perovskites, thin film transistors, tin halide perovskites

## Abstract

Metal halide perovskites (MHPs) are plausible candidates for practical p‐type semiconductors. However, in thin film transistor (TFT) applications, both 2D PEA_2_SnI_4_ and 3D FASnI_3_ MHPs have different drawbacks. In 2D MHP, the TFT mobility is seriously reduced by grain‐boundary issues, whereas 3D MHP has an uncontrollably high hole density, which results in quite a large threshold voltage (*V*_th_). To overcome these problems, a new concept based on a 2D–3D core–shell structure is herein proposed. In the proposed structure, a 3D MHP core is fully isolated by a 2D MHP, providing two desirable effects as follows. (i) *V*_th_ can be independently controlled by the 2D component, and (ii) the grain‐boundary resistance is significantly improved by the 2D/3D interface. Moreover, SnF_2_ additives are used, and they facilitate the formation of the 2D/3D core–shell structure. Consequently, a high‐performance p‐type Sn‐based MHP TFT with a field‐effect mobility of ≈25 cm^2^ V^−1^ s^−1^ is obtained. The voltage gain of a complementary metal oxide semiconductor (CMOS) inverter comprising an n‐channel InGaZnO*_x_* TFT and a p‐channel Sn‐MHP TFT is ≈200 V/V at *V*_DD_ = 20 V. Overall, the proposed 2D/3D core–shell structure is expected to provide a new route for obtaining high‐performance MHP TFTs.

## Graphical abstract

2D–3D core‐shell structure is proposed as a new strategy for overcoming mobility‐threshold voltage (*V*_th_) trade‐off in conventional tin halide perovskite (Sn‐MHP) thin‐film transistors (TFTs). The morphology of fully isolated 3D by 2D Sn‐MHPs generates strong synergy that enables to control *V*_th_ as well as obtain a high field‐effect mobility. The proposed 2D/3D Sn‐MHP TFT demonstrates a high mobility of ≈25 cm^2^ V^−1^ s^−1^.

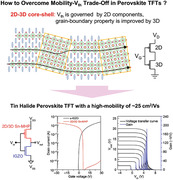

## 1 Advantages of 2D MHPs for Planar‐Type Devices

Metal halide perovskite (MHP) has emerged as a promising candidate for optoelectronic applications, such as photovoltaic devices (PVs) and electroluminescent devices (ELs), due to its excellent photophysical properties and solution processability.^[^ ^1^, ^2^, ^3^ ^]^ MHPs consist of a network of corner‐sharing *BX*_6_ octahedra with the chemical formula *ABX*_3_, where *A* is an organic or inorganic large‐sized cation, *B* is a small‐sized metal cation, and *X* is a halogen anion (e.g., CsPbBr_3_). The *B*–*X*–*B* configuration in a *BX*_6_ octahedron has a bonding angle of 180°, inducing a large dispersion of both the conduction and valence bands originating from superdegeneracy.^[^ ^4^ ^]^ This electronic structure implies that MHPs possess intrinsically outstanding electrical properties. Recently, 2D MHPs have also been extensively studied for PVs and ELs because they provide relatively high photoluminescent quantum yields (PLQYs).^[^ ^2^, ^5^ ^]^ In 2D MHPs, organic cations are intercalated between *BX*_6_ layers, providing a strong exciton confinement effect. Specifically, the electrons and holes are confined in the *BX*_6_ layers (the crystal structures and relevant electronic structures are discussed later in the paper). Nevertheless, the use of 2D MHPs in PVs and ELs faces serious problems. *BX*_6_ layers are energetically isolated by organic cation layers, the electrons and holes can only be transported through the *BX*_6_ octahedra. However, most 2D MHP thin films prefer the c‐axis orientation, whereby *BX*_6_ octahedra connect along with the *a–b* axes. Consequently, 2D MHPs significantly degrade the electrical properties of vertical‐type devices. Sim et al. compared the electrical properties of 2D and 3D MHPs and the performances of EL devices based on these materials. In their experiments, the 3D MHP CsPbBr_2_I realized a very high EL efficiency, but the 2D MHP PEA_2_PbI_4_ achieved a higher PLQY than that of the 3D–CsPbBr_2_I.^[^ ^3^ ^]^ This result implies that 2D MHP is more suited to planar‐type devices, such as thin film transistors (TFTs).

Tin (Sn)‐based MHPs are favored from an environmental perspective because they are less toxic than Pb. Sn‐based MHPs have been extensively studied as a benign alternative to lead (Pb)‐MHPs. They are also prone to the easy formation of Sn vacancies (*V*_Sn_), which are highly demanded in the acceptors of p‐type semiconductors. 2D Sn‐MHPs in p‐type TFTs have been already reported,^[^ ^6^, ^7^, ^8^, ^9^ ^]^ and Zhu et al. recently incorporated 2D PEA_2_SnI_4_ into high‐performance p‐type TFTs with a mobility of 3.5 cm^2^ V^−1^ s^−1^.^[^ ^6^ ^]^ The authors demonstrated the importance of improving the grain‐boundary problems, as emphasized by the high mobility of single‐crystal PEA_2_SnI_4_ TFT (≈40 cm^2^ V^−1^ s^−1^).^[^ ^9^ ^]^ In contrast to their 2D counterparts, 3D Sn‐MHP TFTs have been scarcely reported, probably because the carrier density is difficult to control in 3D Sn‐MHP.

## 2 Concept of High‐Performance MHP TFTs

Motivated by the above background, we herein propose a 2D/3D core–shell structure for high‐performance Sn‐MHP TFTs.

**Figure 1:**
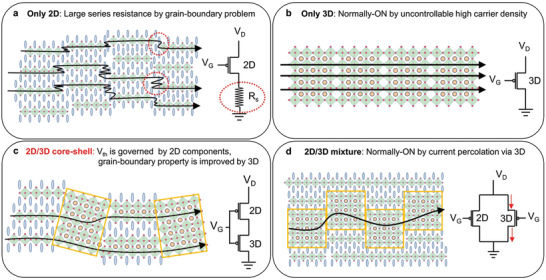
Schematic (cross‐sectional view) of the grain morphologies of 2D, 3D, and 2D/3D mixed Sn‐based MHP thin films and their corresponding equivalent circuits: a) 2D Sn‐MHP, b) 3D Sn‐MHP, c) 2D/3D core–shell Sn‐MHP, and d) 2D/3D mixture Sn‐MHP.

Panels (a,b) of **Figure** **1** demonstrate the drawbacks of conventional 2D and 3D Sn‐MHP TFTs (the equivalent circuits are also shown). These schematics are based on experimentally obtained results. In 2D PEA_2_SnI_4_ TFT, the on‐current is abnormally saturated by the very large series resistance imposed by the grain boundaries (Figure 1a). In 3D Sn‐MHP TFT, only the normally ON state is permitted by the excessive carrier density (Figure 1b). To simultaneously resolve both issues, we propose the 2D/3D core–shell structure shown in Figure 1c. As shown in the equivalent circuit, the current flow in a 2D/3D core–shell structure TFT is inhibited until the 2D component is turned on. That is, the threshold voltage (*V*_th_) is governed by the 2D component regardless of the carrier density of the 3D component. Moreover, the grain‐boundary in this structure is likely superior to those in pure 2D Sn‐MHPs due to the good lattice matching between the 3D and 2D components. It should be noted that a simple 2D/3D mixture cannot meet our proposal because the current only flows through the 3D components (see Figure 1d).

In the following contents, we experimentally examine each case shown in Figure 1. We also show that the addition of SnF_2_ is essential for forming the 2D/3D core–shell structure. The core–shell structure achieved a rather high p‐channel mobility of ≈25 cm^2^ V^−1^ s^−1^ with an on/off ratio of ≈10^6^ and a subthreshold swing (S. S.) of 0.1 V per decade. In a high‐performance complementary metal oxide semiconductor (CMOS) inverter combined by an n‐channel indium–gallium–zinc–oxide (IGZO) TFT and a p‐channel 2D/3D core–shell TFT, the maximum voltage gain was ≈200 V/V at *V*_DD_ = 20 V. The performance of this CMOS inverter is comparable to that of an n‐channel IGZO TFT combined with a p‐channel formed from low‐temperature polysilicon.^[^ ^10^ ^]^

## 3 Results and Discussion

### 3.1 Crystal and Electronic Structures of 2D‐PEA_2_SnI_4_ and 3D‐FASnI_3_ and the TFT Performances

**Figure 2:**
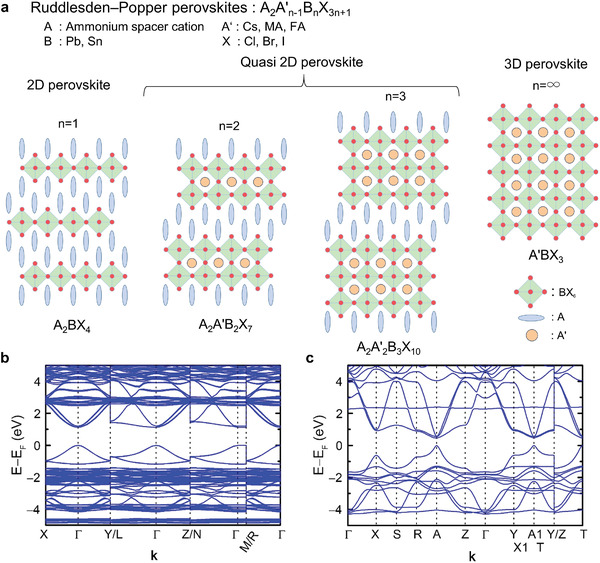
Comparison of the crystal and electronic structures of the 2D and 3D metal halide perovskites: a) crystal structures of 2D, quasi‐2D, and 3D MHPs; band structures of b) 2D PEA_2_SnI_4_ and c) 3D FASnI_3_.

**Figure** **2**a compares the crystal structures of the 2D and 3D MHPs with the chemical formulas of *A*_2_ *A*′*_n_*_–1_ *B_n_X*_3_ *_n_*_+1_ and *A*′*BX*_3_, respectively. Here, *A*, *A*′, *B*, and *X* denote the ammonium spacer cation, organic or metal cation, metal cation, and halogen anion, respectively. In this study, phenethyl ammonium (PEA), formamidinium (FA), Sn, and I correspond to *A*, *A′*, *B*, and *X*, respectively. The 2D MHPs can be further categorized into a pure 2D phase (*A*_2_ *BX*_4_) and a quasi‐2D phase (*A*_2_ *A′_n–_*_1_ *B_n_X*_3_ *_n_*_+1_, *n* >1). The crystal structure suggests that with the increase in *n*, the physical property of quasi‐2D MHP approaches that of 3D MHP. The calculated band structures indicate that both 2D PEA_2_SnI_4_ and 3D FASnI_3_ have large dispersions at the valence band maximum (Figures 2b,c); the effective masses are given in **Table** **1**. The effective hole mass was smaller in 2D PEA_2_SnI_4_ than in FASnI_3_, reflecting the shorter Sn–I distance in PEA_2_SnI_4_ (3.13 Å) than in FASnI_3_ (3.2 Å).

**Table 1:** Effective masses of the electrons and holes in PEA_2_SnI_4_ and FASnI_3_

	2D‐PEA_2_SnI_4_		3D‐FASnI_3_	
	Line	m*	Line	m*
*m*_h_	Γ‐*X*	0.04	*A*1–*X*1	0.06
*m*_e_	Γ‐*X*	0.03	*A*1–*X*1	0.04

The calculated densities of the states indicate that the hybridized Sn 5s and I 5p states controlling the valence band maximum reduced the effective hole mass in Sn‐MHPs (Figure S1, Supporting Information).^[^ ^11^ ^]^ In other words, the p‐type conduction in Sn‐MHPs largely depends on the connectivity of the [SnI_6_]^4−^ polyhedra, which is consistent with the c‐axis direction of the band structure obtained by the density functional theory; note the highly localized flat band in the Γ‐M region of PEA_2_SnI_4_ (Figure 2b). Thus, it was inferred that the p‐type conduction properties in 2D PEA_2_SnI_4_ depend on the crystal growth orientation and/or the structures of electronic devices. For example, ELs and PVs are vertical‐type devices, while TFTs are planar‐type devices.

**Figure 3:**
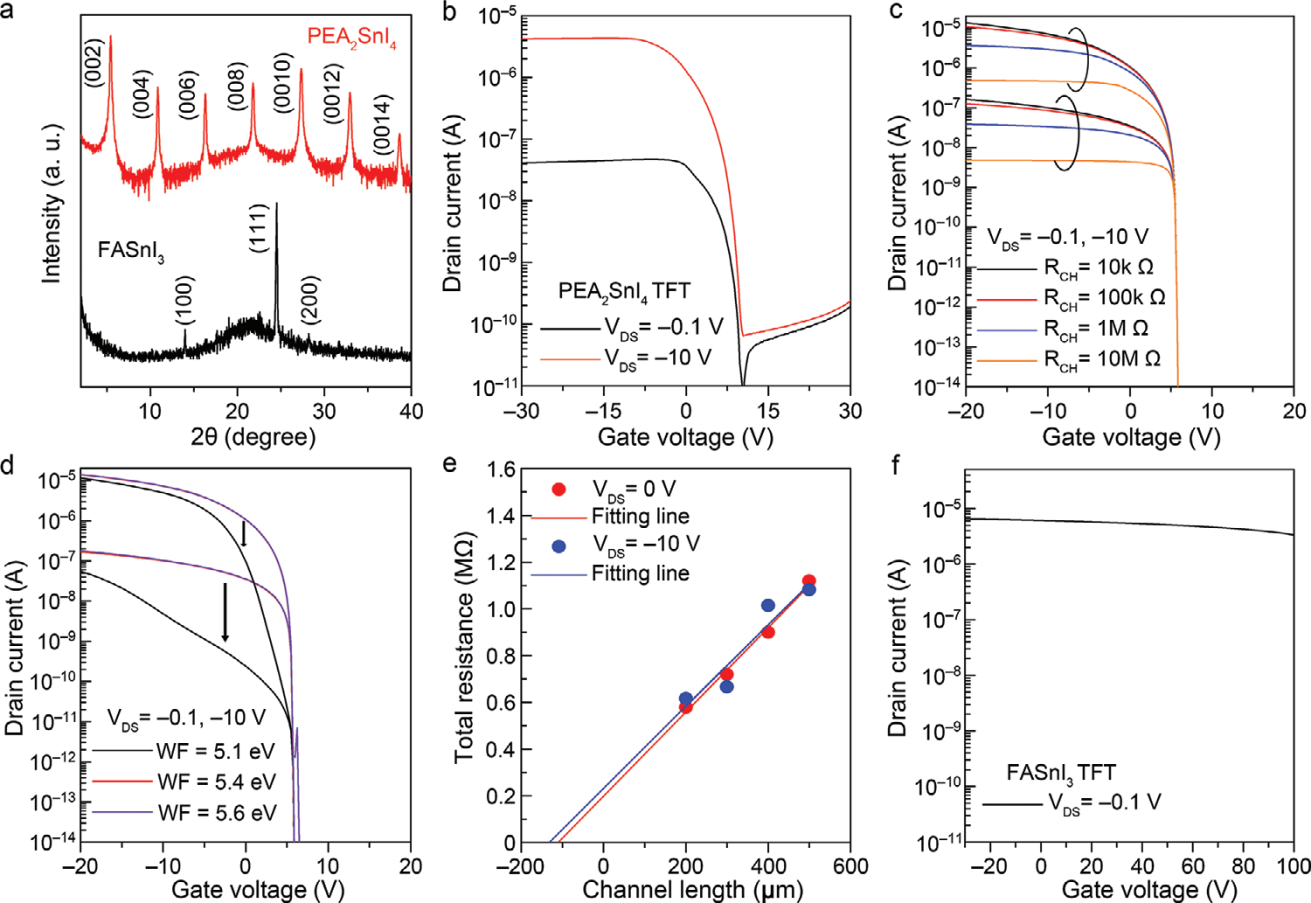
TFT performances of 2D PEA_2_SnI_4_ and 3D FASnI_3_ TFTs. a) Comparison of the XRD patterns; b) transfer curve of 2D PEA_2_SnI_4_ TFT; device simulation results of the models for c) various series resistances in the channel layer and d) various Schottky barrier heights between the source/drain electrodes and active layer; e) total resistance of the 2D PEA_2_SnI_4_ active layer determined by the transmission line method; f) transfer curve of 3D FASnI_3_ TFT.

Through the conducted out‐of‐plane X‐ray diffraction (XRD) and rocking curve analyses, we further confirmed that 2D PEA_2_SnI_4_ thin films prefer c‐axis oriented growth (see **Figure** **3**a; Figure S2, Supporting Information). Although high p‐type conduction was anticipated because the corner‐sharing SnI_6_ layers are parallel to the current flow direction in TFTs, the 2D PEA_2_SnI_4_‐based TFT failed to deliver satisfactory performance because it suffered from abnormal on‐current saturation (Figure 3b). Two primary reasons can explain this phenomenon: the (i) series resistance and the (ii) Schottky barrier between the metal electrode and active layer. These potential causes were investigated in the device simulations of the two cases. Figure 3c shows the effect of the series resistance on the simulated TFT performance. Obviously, the on‐current was saturated when the series resistance became very high, but no on‐current saturation behaviors appeared in the simulated results of the Schottky barrier model (Figure 3d). It was concluded that the series resistance in the 2D PEA_2_SnI_4_ TFT is excessively large. We consider that identifying this cause can pave the way for obtaining high‐performance Sn‐MHP TFTs.

The origin of the series resistance in the 2D PEA_2_SnI_4_ TFT was measured using the transmission line method (TLM). When a gate voltage was applied, the slope of the total resistance usually decreased with the accumulation of carriers. On the contrary, the total resistance in the TLM analysis (see Figure 3e) was unchanged after applying the gate bias. Furthermore, the obtained contact resistance was ≈0.2 MΩ, which is negligibly smaller than the channel resistance. The quite large series resistance was attributed to the grain boundaries in the 2D PEA_2_SnI_4_. As discussed earlier, the connectivity of the [SnI_6_]^4–^ polyhedra was the criterion of the p‐type conduction in Sn‐MHPs. This criterion should also be satisfied at the grain boundaries. However, it seems that 2D PEA_2_SnI_4_ is structurally less favorable than 3D Sn‐MHP with regard to making grain connections through [SnI_6_]^4−^ (see Figure 1a). Recently, Zhu et al. reported that improving the grain‐boundary in PEA_2_SnI_4_ largely improves the TFT mobility from 0.21 to 3.5 cm^2^V^−1^ s^−1^.^[^ ^6^ ^]^

To compare the electrical properties of the 2D and 3D Sn‐MHPs, we fabricated FASnI_3_ TFT and plotted its transfer curve (see Figure 3f). The on‐current was much larger in the 3D FASnI_3_ TFT than in the 2D PEA_2_SnI_4_ TFT, suggesting that 3D FASnI_3_ is less affected by the grain‐boundary problem. However, the 3D FASnI_3_ TFT was not turned off even under a gate bias of 100 V. As discussed above (Figure 1b), the hole density in the 3D FASnI_3_ was excessively raised by the readily formed *V*_Sn_ defects.^[^ ^12^ ^]^

### 3.2 2D/3D Core–Shell Structure and TFT Performances

Section 3.1 discusses the various drawbacks of 2D PEA_2_SnI_4_ and 3D FASnI_3_. The electronic structure and hole concentration of 2D PEA_2_SnI_4_ are suitable for TFT channels, but the hole transport is seriously deteriorated by the poor grain‐boundary properties. On the contrary, 3D FASnI_3_ possesses better grain‐boundary properties than 2D PEA_2_SnI_4_ but is limited to the normal ON condition owing to the uncontrollable high hole density.

**Figure 4:**
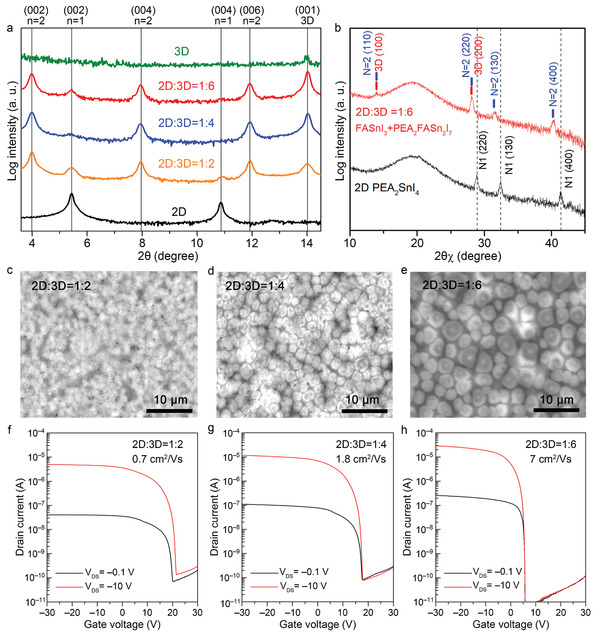
Characterization of the thin films with the 2D/3D core–shell structure synthesized from a mixed precursor solution of 3D FASnI_3_ and 2D PEA_2_SnI_4_ (top, center rows) and the TFT performances (bottom row): a) out‐of‐plane XRD patterns, b) in‐plane XRD patterns, SEM images of the thin films with 2D:3D mixing ratios of c) 1:2, d) 1:4, and e) 1:6; transfer curves of the TFTs fabricated from thin films with 2D:3D mixing ratios of f) 1:2, g) 1:4, and h) 1:6.

Hereafter, we demonstrate that combining the 2D and 3D materials in an appropriate configuration can resolve the conflicting drawbacks in 2D and 3D MHPs (Figure 1c). First, various precursor solutions were prepared by mixing precursor solutions of 2D PEA_2_SnI_4_ and 3D FASnI_3_ at different ratios (1:2, 1:4, and 1:6). Then, out‐of‐plane XRD measurements were performed (**Figure** **4**a), and they revealed that all the films were mixed phases of 2D PEA_2_SnI_4_, quasi‐2D PEA_2_FASn_2_I_7_, and 3D FASnI_3_. The quasi‐2D PEA_2_FASn_2_I_7_ and 3D FASnI_3_ phases dominated, and the 3D‐related peak became stronger with the increase in the mixing ratio of the 3D content. Meanwhile, the in‐plane direction of the crystallinity determined the hole transport because it aligned with the current flow in TFTs. Only the (*hk*0)‐plane‐related peaks appeared in the in‐plane XRD patterns (Figure 4b). These peaks originated from the periodically aligned SnI_6_ polyhedra, implying that hole transport occurred via the 2D and 3D grains interconnected by the corner‐sharing SnI_6_ polyhedra.

Note that the preferential orientation of the 3D FASnI_3_ changed from (111) to (001) in the 2D/3D mixed thin films (c.f. Figures 3a and 4a). Based on these observations, we considered whether quasi‐2D PEA_2_FASn_2_I_7_ and 3D FASnI_3_ can form a natural superlattice structure. In the XRD patterns of Figure 4b, the peak around 28.1° originated from two components: the (220) plane of quasi‐2D PEA_2_FASn_2_I_7_ and the (200) plane of 3D FASnI_3_, indicating the close d‐spacing values of the two materials. The in‐plane XRD patterns of different 2D/3D ratios were also compared, as shown in Figure S3, Supporting Information. The d‐spacing values calculated from the crystal structures of quasi‐2D PEA_2_FASn_2_I_7_ and 3D FASnI_3_ were found to be 3.05 and 3.15 Å, respectively. Therefore, it is suggested that the preferentially formed quasi‐2D PEA_2_FASn_2_I_7_ works as the seed layer for 3D FASnI_3_ (see the expected structure in Figure S4d, Supporting Information). We confirmed that the preferential orientation of the 3D FASnI_3_ changed from (111) to (001) by the addition of a small amount of 2D PEA_2_SnI_4_ (2 mol%), as shown in Figures S4a,b, Supporting Information. This result implies that 2D PEA_2_SnI_4_ largely contributes to the crystal growth of 3D Sn‐MHPs. This natural superlattice structure may explain why the quasi‐2D PEA_2_FASn_2_I_7_ and 3D FASnI_3_ phases dominated the other phase at all the mixing ratios. Note that when the mixing ratio was 1:6, the chemical composition corresponded to the chemical formula PEA_2_FA*_n−_*_1_Sn*_n_* I_3_ *_n_*_+1_ (*n* = 7). There have already been reports on the superlattice structure in quasi‐2D Sn‐MHP thin films.^[^ ^13^ ^]^

The scanning electron microscope (SEM) images (Figures 4c–e) clarify the formation of a 2D/3D core–shell structure in the sample with a 2D:3D mixing ratio of 1:6 (different mixing ratios with different scales were provided, as shown in Figure S5, Supporting Information). As the microcrystals were spatially isolated and their size increased with the increase in 3D content, they were assigned to the 3D FASnI_3_. The microcrystals were further investigated by an electron probe microanalyzer (EPMA). Judging from the chemical formulas of the 2D and 3D phases, the composition ratio of Sn to I was smaller in the 2D phases than in the 3D phase. Thus, we investigated the Sn/I ratio in the regions of the microcrystal and its boundary. The iodine content was lower in the microcrystal region than in the boundary region. The Sn/I ratio difference between the microcrystal interior and boundary was ≈0.06, which is consistent with the difference between quasi‐2D (*n* = 2) and pure 3D. We concluded that the microcrystals originated from 3D FASnI_3_.

To confirm the effect of the 2D/3D core–shell structure, we compared the performances of the TFTs fabricated from thin films with different 2D:3D mixing ratios (see Figure  4f–h). The on‐current saturation problem was fully solved by the 2D/3D core–shell structure, which boosted the field‐effect mobility to 7 cm^2^ V^−1^ s^−1^. Moreover, we can see that *V*_th_ can be controlled even though the majority component is the 3D Sn‐MHP. This result demonstrates the effectiveness of the proposed strategy shown in Figure 1c. However, we found that microstructural analyses are rather difficult for Sn‐MHP because Sn‐MHP is readily decomposed by X‐ray and electron beams. As shown in Figure S6a, Supporting Information, the repeated XRD measurement resulted in the decomposition of the 2D Sn‐perovskites; we could see that a PEAI phase emerged after the decomposition. The decomposition of Sn‐perovskite was also confirmed through X‐ray photoemission spectroscopy (XPS) measurements. Many researchers have discussed the oxidation states of Sn^2+^/Sn^4+^.^[^ ^14^, ^15^, ^16^ ^]^ However, as shown in Figure S6b, Supporting Information, two components exist for iodine, implying that the peak separation of Sn is also possibly caused by the decomposition rather than oxidation states of Sn. Owing to such a decomposition problem in Sn‐MHPs, the transmission electron microscopy (TEM) observation was unsuccessful. As shown in Figure S7, Supporting Information, the obtained TEM image indicates that the Sn‐perovskite layer was fully converted into an amorphous phase.

### 3.3 Significance Effect of the SnF_2_ Additive

In Section 3.2, we demonstrated the validity of the 2D/3D core–shell structure with regard to obtaining high‐performance Sn‐perovskite TFTs. In fact, all the Sn‐perovskite samples shown in Figures 3 and 4 were synthesized with the SnF_2_ additive. Hereafter, we show the critical role of the SnF_2_ additive for obtaining the 2D/3D core–shell structure. The SnF_2_ additive has been widely used in the preparation of Sn‐based MHPs. Many studies have reported that SnF_2_ additives suppress *V*_Sn_ defects.^[^ ^14^, ^15^, ^16^ ^]^ Unlike the relatively stable Pb‐based MHPs, Sn‐based MHPs readily form *V*_Sn_ because the standard redox potential of Sn^2+^/Sn^4+^ is much lower (+0.15 V) than that of Pb^2+^/Pb^4+^;(+1.67 V), implying that Sn^2+^ is easily oxidized. To prevent this intrinsic disadvantage of Sn‐MHPs, researchers usually add SnF_2_ as an effective control method of Sn^2+^ oxidation. The SnF_2_ additive is known to elevate the formation energy of *V*_Sn_.^[^ ^15^, ^16^ ^]^

**Figure 5:**
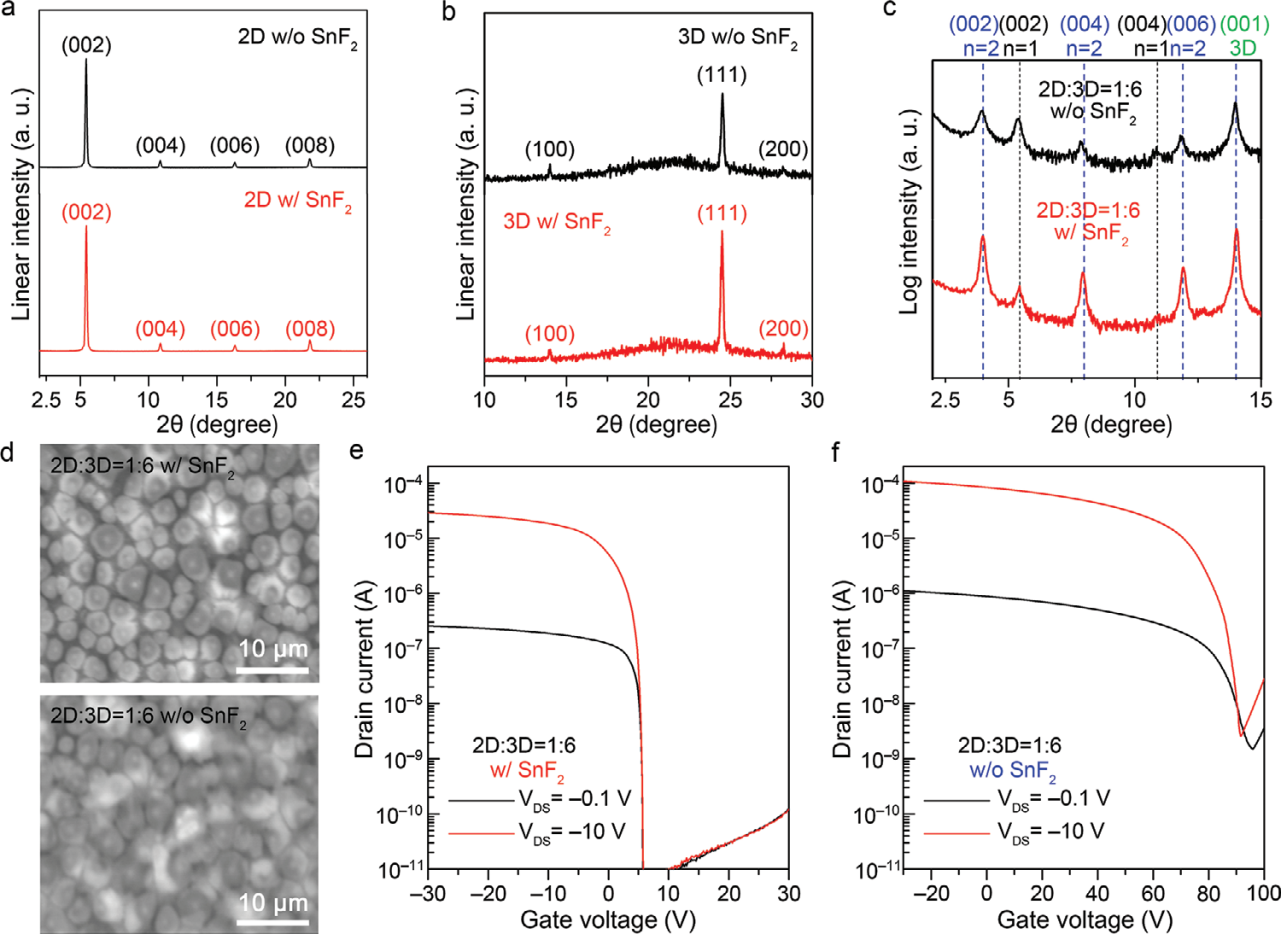
Role of the SnF_2_ additive in Sn‐MHPs thin films: out‐of‐plane XRD patterns of a) 2D PEA_2_SnI_4_, b) 3D FASnI_3_, and c) 2D/3D thin films; d) SEM images of 2D/3D thin films; transfer curves of the 2D/3D TFTs e) w/and f) w/o SnF_2_ additive. The mixing ratio of the thin films was 2D:3D = 1:6, and 2 mol% SnF_2_ was added. In (c), *n* = 1 and *n* = 2 correspond to PEA_2_SnI_4_ and PEA_2_FASn_2_I_7_, respectively.

We found a new role for SnF_2_ in the preferential crystallization of 3D MHPs rather than 2D MHPs. Panels (a,b) of **Figure** **5** show the XRD patterns of the PEA_2_SnI_4_ and FASnI_3_ thin films, respectively, with and without the 2 mol% SnF_2_ additive. The crystallinity was significantly affected by the SnF_2_ additive only in the 3D FASnI_3_. This feature is highly relevant to the formation of the quasi‐2D phase (*n* >1) crystal structure, in which the 3D FASnI_3_ component is sandwiched between PEA cations (Figure 2a). As clarified in the XRD patterns shown in Figure 5c, the intensity of the peak related to the pure 2D phase (*n* = 1) decreased after adding SnF_2_. It was concluded that SnF_2_ plays a critical role in forming 3D‐FASnI_3_ crystals.

The effect of the SnF_2_ additive on the FASnI_3_ crystallization was crucial to the formation of the 2D/3D core–shell structure. In the thin film without the SnF_2_ additive, the 3D FASnI_3_ microcrystals were randomly connected, whereas the film with the SnF_2_ additive showed a rapidly crystallized 3D FASnI_3_ phase with clear separation between the 2D and 3D phases (see the SEM images in Figure 5d). It should also be noted that 2 mol% is the optimum amount of the SnF_2_ additive, where with the increase in the SnF_2_ content beyond 2 mol%, the TFT mobility was gradually deteriorated (see Figure S8, Supporting Information). The morphology significantly affected the TFT performance. Panels (e) and (f) (Figure 5) display the transfer curves of the 2D/3D TFTs with phase‐separated and the randomly connected morphologies, respectively. The randomly connected TFT yielded a large *V*_th_ (90 V), whereas in the TFT with the 2D/3D core–shell structure, *V*_th_ was reduced to 5 V.

### 3.4 Significance of Removing the Residual Solvent

**Figure 6:**
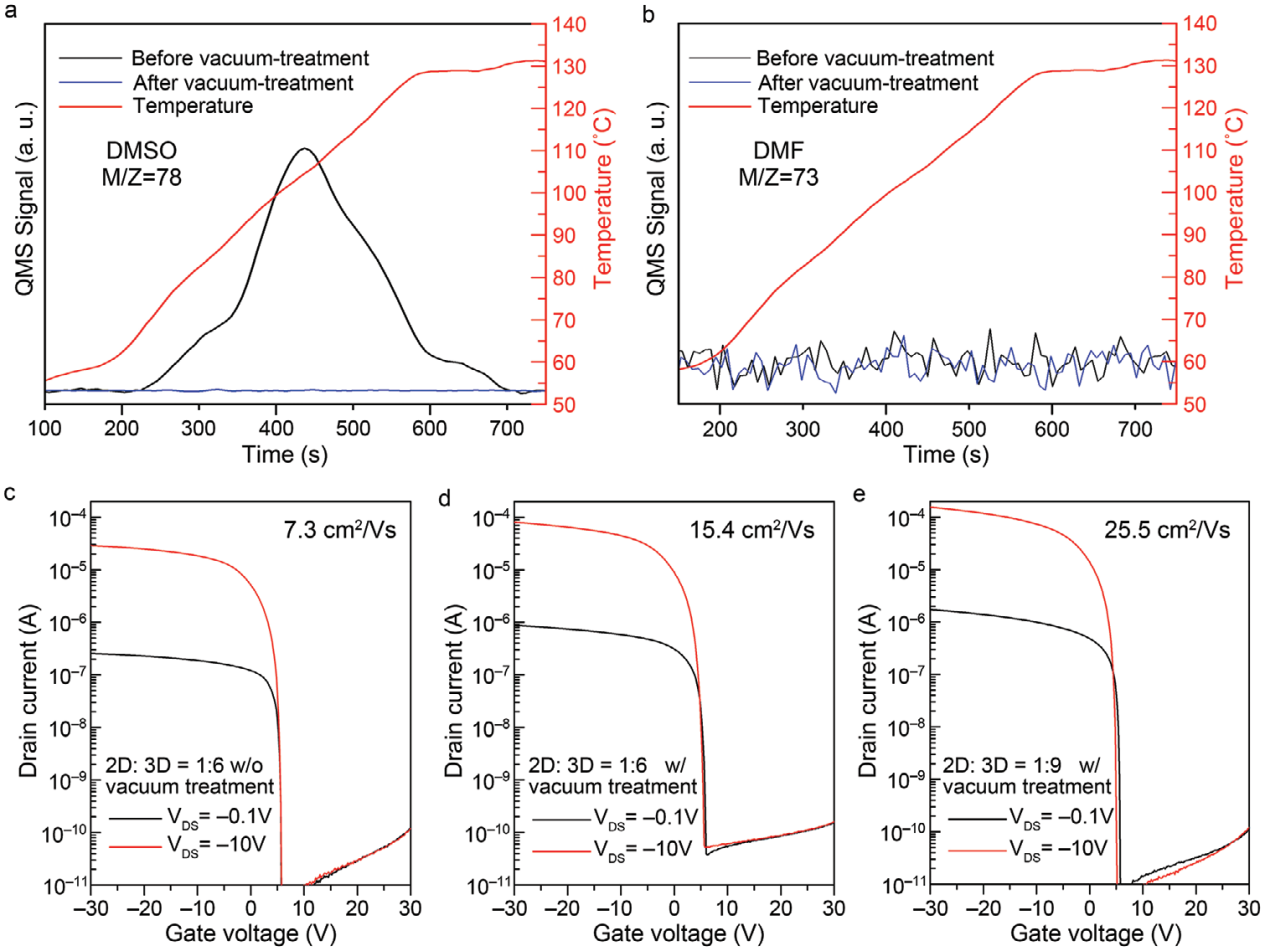
Effect of vacuum treatment on Sn‐MHPs: Thermal desorption spectra of the 2D/3D thin films (2D:3D = 1:6) w/and w/o vacuum treatment for the a) mass number 78 (DMSO) and b) mass number 73 (DMF); c) TFT performances of the 2D/3D TFTs (2D:3D = 1:6) w/o vacuum treatment; TFT performances of the vacuum treated TFTs with different 2D/3D ratios: d) 2D:3D = 1:6 and e) 2D:3D = 1:9.

In the previous subsections, we revealed that the 2D/3D core–shell structure significantly improved the TFT performance. This finding suggests that the crystallinity and grain‐boundary properties along the in‐plane direction are critical for p‐type conduction. When analyzing the Sn‐MHP thin films, we found that the as‐fabricated films included a residual solvent of dimethyl sulfoxide (DMSO). Thermal desorption spectroscopy (TDS) measurements (**Figures** **6**a,b) were performed, and they clarified that DMSO, rather than dimethylformamide (DMF), remained in the thin films because DMSO has a higher boiling temperature than that of DMF (≈190 °C vs ≈150 °C). In this study, the mixing ratio of DMSO/DMF (1:1) was used because DMSO is essential for obtaining the core–shell structure (see the Experimental Section for details). As shown in Figure S9, Supporting Information, the 3D microcrystals drastically shrank without DMSO. Different solvents evidently affect the film morphology and crystallinity of solution‐processed thin films.^[^ ^17^, ^18^ ^]^ It has been reported that less volatile solvents such as DMSO within perovskite films can assist in mass transportation and diffusion, thus leading to improved film quality.^[^ ^6^, ^19^ ^]^

To fully utilize solvent engineering and reduce the negative effects of the nonvolatile solvents, we stored the MHP thin films in vacuum (10^−6^ Pa for 6 h) to facilitate the escape of DMSO from the film. As shown in Figure 6a, the peak in the TDS of the mass number 78 (corresponding to DMSO) disappeared by the vacuum storage. However, it was confirmed that post‐annealing treatments also enable the elimination of DMSO but readily destroy the Sn‐MHP phase due to poor thermal stability.^[^ ^20^ ^]^ Thus, it was revealed that the room‐temperature vacuum treatment is the most appropriate method for removing undesired chemicals from MHP thin films. As shown in Figure 6c,d, it is obvious that the vacuum treatment improved the field‐effect mobility of the 2D/3D core–shell TFT by twofold. Consequently, we achieved a much higher field‐effect mobility of 25.5 cm^2^ V^−1^ s^−1^ by using a slightly higher 3D content (2D:3D = 1:9) with the vacuum treatment (see Figure 6e). The TFT parameters are summarized in detail in **Table** **2**. The device performance of the present work was compared with the recent results from other groups in Table S2, Supporting Information.

**Table 2:** TFT properties of the 2D and 2D/3D core–shell structure after vacuum storage

	*V*_th_ (V)	*μ*_Linear_ (cm^2^ V^−1^ s^−1^)	S. S. (V per decade)	*I*_on_/*I*_off_
PEA_2_SnI_4_	2.48	N/A	0.77	>10^4^
2D:3D = 1:6	4.83	7.3	0.10	>10^5^
2D:3D = 1:6				
w/vacuum treatment	5.04	15.4	0.19	>10^6^
2D:3D = 1:9				
w/vacuum treatment	5.24	25.5	0.09	>10^6^

Most of the Sn‐MHP TFTs somewhat exhibited serious hysteresis (see Figure S10, Supporting Information). We also noticed a large negative gate bias effect in the fabricated Sn‐MHP TFTs. We thus surmised that hysteresis occurs when positively charged species are captured in the gate insulator (GI). For instance, protons (H^+^) from organic precursors might diffuse into GI.^[^ ^21^ ^]^ Matsushima et al. reported that such hysteresis behaviors can be significantly improved by modifying the GI interface.^[^ ^8^ ^]^

### 3.5 CMOS Application of the 2D/3D Core–Shell Structure and Amorphous IGZO TFT

**Figure 7:**
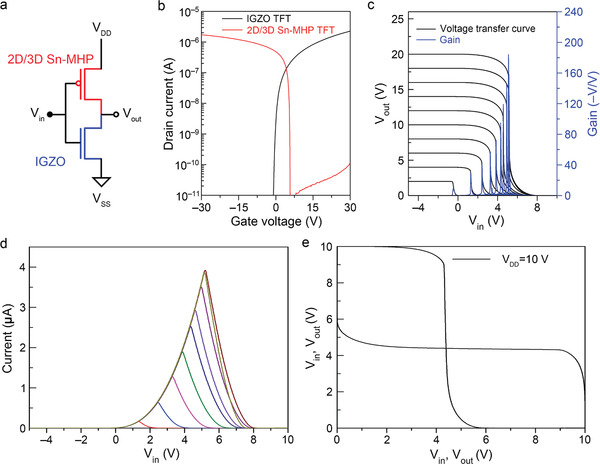
Characteristics of the inverter fabricated from an n‐type a‐IGZO and p‐type 2D/3D (2D/3D = 1:9) TFTs: a) Schematic of the inverter; b) transfer curves; c) voltage transfer curve and voltage gain of the inverter; d) leakage current of the inverter; and e) butterfly plot of the inverter.

By applying the proposed scheme and sequential posttreatment, a highly reproducible p‐type TFT based on a 2D/3D core–shell perovskite was fabricated. The TFT delivered an excellent field‐effect mobility of 25.5 cm^2^ V^−1^ s^−1^ with a threshold voltage of 5.0 V. This result is electrically promising for constructing inverters with n‐type a‐IGZO TFTs (field mobility ≈20 cm^2^ V^−1^ s^−1^). The channel length and width should satisfy (*W*/*L*)_p_ = *μ*_n_/*μ*_p_ × (*W*/*L*)_n_, where (*W*/*L*)_p_ and (*W*/*L*)_n_ are the width‐to‐length ratios of the p‐channel and n‐channel, respectively, and *μ*_n_/*μ*_p_ is the ratio of the mobilities in the n‐channel and p‐channel.^[^ ^22^ ^]^ If the mobilities of the p‐channel and n‐channel are similar, the channels need not be fabricated with the distinguished patterns. Thus, the process could be simplified. Here, we constructed a high‐performance complementary inverter with the same channel width to channel length ratio of both channels. **Figure** **7**b shows the corresponding transfer curves of the p‐channel and n‐channel at *V*_DS_ = ±0.1 V, and Figure 7c depicts the voltage transfer curves of the fabricated inverter at different *V*_DD_. The inverter produced the proper high (1) and low (0) logic signals and gave ideal output characteristics with a high voltage gain (≈70 V/V at *V*_DD_ = 10 V, ≈200 V/V at *V*_DD_ = 20 V). From the *I*_DD_ versus *V*_in_ plot (Figure 7d), the current flow was determined as 2 µA at *V*_DD_ = 10 V, confirming the low power consumption in this device. From the butterfly plot (Figure 7e), the noise margin high (NM_H_) and noise margin low (NM_L_) were calculated as 4.3 and 3.72, respectively. The total noise margin, obtained as the minimum of NM_H_ and NM_L_ (i.e., 3.72), was 74% of the ideal value (*V*_DD_/2). Overall, the invertor fabricated from the 3D/2D core–shell structure and a‐IGZO TFT demonstrated excellent performance.

## 4 Summary

The proposed strategy was designed to solve the drawbacks of conventional Sn‐MHPs. After elucidating the physical properties of the MHP (electronic structure, crystal structure, and morphology), we reached the following conclusions.

(1) The *c*‐axis‐oriented 2D MHP thin film is intrinsically suitable for planar‐type devices, such as TFTs, because the carrier conduction path of the SnI_6_ layer matches the current flow direction in TFTs.
(2) Mobility‐threshold voltage trade‐off relation was clarified in Sn‐MHP TFTs; 2D PEA_2_SnI_4_ TFT exhibits a poor performance owing to serious grain boundary problems, whereas 3D FASnI_3_ TFT suffers from normally‐ON state owing to uncontrollable high hole density.
(3) The proposed 2D/3D core–shell structure generates a strong synergy that solves the conflicting drawbacks in 2D PEA_2_SnI_4_ and 3D FASnI_3_ TFTs. The morphology of fully isolated 3D by 2D enables to control *V*_th_ as well as obtain a high field‐effect mobility.
(4) The SnF_2_ additive is critical for obtaining 2D/3D core–shell structures, and the vacuum treatment eliminates the residual DMSO in thin films. Consequently, these treatments boosted the TFT mobility from 7 to 25 cm^2^ V^−1^ s^−1^.
(5) A high‐performance CMOS inverter with a high voltage gain of 200 V/V at V_D__D_ = 20 V was realized by combining p‐channel 2D/3D TFTs with an n‐channel IGZO TFT. This performance is the best reported so far for CMOS using Sn‐MHP TFTs.

The present strategy is applicable to various solution‐derived semiconductor systems, paving the way for future flexible and printable electronic devices.

## 5 Experimental Section

### Density Functional Theory (DFT) Calculation

DFT calculations were performed using the generalized gradient approximation with the Perdew–Burke–Ernzerhof functional and projected augmented plane wave method implemented in the Vienna ab initio simulation program (VASP).^[^ ^23^, ^24^, ^25^ ^]^ The initial structural parameters of 2D PEA_2_SnI_4_ and 3D FASnI_3_ were taken from previous papers,^[^ ^23^, ^26^ ^]^ respectively. The band structure, effective mass, and density of the states were calculated after relaxing the structure by minimizing its total energy and force. The plane wave basis energy cutoff was set to 600 eV, and FASnI_3_ and PEA_2_SnI_4_ were structurally relaxed on 8 × 8 × 8 and 4 × 4 × 2 *k*‐point grids, respectively.

### Preparation and Characterization of the MHP Thin Films

All the chemicals were purchased from Sigma–Aldrich and utilized without further purification. 0.3 m PEA_2_SnI_4_ was obtained by dissolving PEAI and SnI_2_ in a DMF:DMSO = 1:1 volume ratio at a molar ratio of 2:1. 0.3 m FASnI_3_ was obtained by dissolving FAI and SnI_2_ in a DMF:DMSO = 1:1 volume ratio at a molar ratio of 1:1. For the TFT fabricated by FASnI_3_, slight PEA_2_SnI_4_ (less than 5 mol%) was added to the FASnI_3_ precursor for better film quality. 0.3 m 2D:3D = 1:2, 1:4, and 1:6 precursors were obtained by mixing 0.3 m PEA_2_SnI_4_ and 0.3 m FASnI_3_ in a DMF:DMSO = 1:1 volume ratio at the molar ratios of 1:2, 1:4, and 1:6, respectively. 2 mol% SnF_2_ was incorporated in each precursor above. 0.3 m 2D:3D = 1:6 precursors with 2%, 4%, and 6% SnF_2_ were obtained by mixing 0.3 m PEA_2_SnI_4_ and 0.3 m FASnI_3_ in a DMF:DMSO = 1:1 volume ratio at a molar ratio of 1:6 with additional 2 mol%, 4 mol%, and 6 mol% of SnF_2_. MHP thin films were prepared by spin coating at 5000 rpm for 60 s. Each MHP film was annealed in a glove box (Ar ambient) at 100 °C for 10 min. For the MHP films with vacuum treatment, the MHP films were first dried in the glove box (Ar ambient) at 100 °C for 10 min and then stored in vacuum (10^−6^ Pa) for 6 h. The crystal structures of the MHP films were characterized by both out‐of‐plane and in‐plane X‐ray diffraction spectroscopy (SmartLab, Rigaku). MHP films with and without vacuum treatment were prepared for TDS measurements. The morphology and composition of the MHP films were provided by SEM and EPMA.

### Fabrication, Characterization, and Simulation of the Device

Bottom‐gate and bottom‐contact TFTs were fabricated on a SiO_2_/p++‐silicon substrate. The thickness of the thermally grown SiO_2_ was 150 nm, and the sequential 40 nm platinum source/drain electrodes were deposited through a shadow mask. The channel was 1000 µm wide and 500 µm long. The substrates with their electrodes were then subjected to UV–ozone for 30 min. After the UV–ozone treatment, the channels were spin‐coated at 5000 rpm for 60 s in an Ar‐filled glove box. The MHP devices were annealed and vacuum treated as described in ′Preparation and Characterization of the MHP Thin Films′. Each device was passivated by 30‐nm‐thick *N,N*′‐Di(1‐naphthyl)‐*N,N*′‐diphenyl‐(1,1′‐biphenyl)‐4,4′‐diamine (NPD) to avoid air effects during the process. All the devices were measured at *V*_DS_ = −0.1 V, −10 V under vacuum conditions (1 × 10^−3^ Pa) by a KEYSIGHT B1500A. The complementary circuits were measured at *V*_DD_ = 2–20 V, where *V*_in_ was swept from 10 to −5 V. The device characteristics were calculated by a device simulator (Atlas, Silvaco Inc.). The simulation configuration was similar to that of the fabricated device. The field‐effect linear mobility of the device was calculated as $\mu_{\mathrm{Linear}}=\frac{L}{WC_{ox}V_{\mathrm{DS}}}\;g_{m_{\max}}$, where$\;g_{m_{\max}}$ is the maximum transconductance, *C*_ox_ is the unit area capacitance of the gate dielectric, *V*_DS_ is the drain voltage, and *W* and *L* are the channel width and length, respectively. The threshold voltage of the device was estimated as the gate voltage at the current level given by $\frac{\text{Channel}\;\text{Width}(W)}{\text{Channel}\;\text{Length}(L)}\;\times 10\;\mathrm{nA}$.

## Conflict of Interest

The authors declare no conflict of interest.

## Supporting information

Supporting Information

## Data Availability

The data that support the findings of this study are available on request from the corresponding author.
