# Supplementary material for: High‐Performance P‐Channel Tin Halide Perovskite Thin Film Transistor Utilizing a 2D–3D Core–Shell Structure
Source: Adv Sci (Weinh). 2021 Dec 19;9(5):2104993. doi: 10.1002/advs.202104993 (PMC8844482; doi:10.1002/advs.202104993)
Supplement: Supplementary file 1 — Supporting Information [file ADVS-9-2104993-s001.pdf]

ADVANCED SCIENCE
Open Access

Supporting Information

for *Adv. Sci.,* DOI: 10.1002/advs.202104339

High-Performance P-channel Tin Halide Perovskite Thin Film Transistor Utilizing a 2D-3D Core–shell structure

Junghwan Kim, Yu-Shien Shiah, Kihyung Sim, Soshi Iimura, Katsumi Abe, Masatake Tsuji, Masato Sasase, Hideo Hosono**

Supporting Information

High-Performance P-channel Tin Halide Perovskite Thin Film Transistor Utilizing a 2D-3D Core–shell structure

Junghwan Kim,#, Yu-Shien Shiah#, Kihyung Sim, Soshi Iimura, Katsumi Abe, Masatake Tsuji, Masato Sasase, Hideo Hosono**

Table S1. Comparison of the XRD peak parameters of the 2D/3D thin films with and without SnF_2.

2D:3D = 1:6 w/o SnF_2					2D:3D = 1:6 w/ SnF_2				
Phase	2 theta (deg)	Miller index	FWHM (deg)	Size (nm)	Phase	2 theta (deg)	Miller index	FWHM (deg)	Size (nm)
N1	5.46	002	0.237	35.06	N1	5.46	002	0.344	24.16
N1	--	004	--	--	N1	--	004	--	--
N2	4.02	002	0.258	32.19	N2	3.98	002	0.174	47.73
N2	7.96	004	0.231	36.02	N2	7.96	004	0.197	42.24
N2	11.92	006	0.207	40.32	N2	11.92	006	0.19	43.92
3D	14.04	001	0.15	55.75	3D	14.04	001	0.128	65.34

Table S2. Comparison of the recently reported p-type MHP TFTs.

Materials	Dielectric layer	V_{th} (V)	μ_{Linear} (cm^2/Vs)	$\mu_{Saturation}$ (cm^2/Vs)	S. S. (V/decade)	I_{on}/I_{off}	Refs.
PEA_2SnI_4: $FASnI_3$ =1:6	SiO_2	5.04	15.41	12.71	0.19	$>10^4$	**This work**
PEA_2SnI_4: $FASnI_3$ =1:9	SiO_2	5.24	25.46	22.02	0.09	$>10^4$	**This work**
PEA_2SnI_4	SiO_2	7.3	N/A	3.51	0.8	$>10^6$	[1]
PEA_2SnI_4	SiO_2	N/A	N/A	0.6	N/A	$>10^4$	[2]
PEA_2SnI_4	CYTOP	−22	N/A	12~15	0.8	10^6	[3]
$(4Tm)_2SnI_4$	SiO_2	<−20	N/A	2.32	~10	$>10^5$	[4]
PEA_2SnI_4/ Semi-CNT	SiO_2	25	N/A	1.51	2.1	$>10^5$	[5]
PEA_2SnI_4/ CuI	SiO_2	7.0	N/A	2.61	0.5	$>10^6$	[6]
$FASnI_3$/ PEA_2SnI_4	Al_2O_3/ PMMA	2.8	N/A	0.21	N/A	10^4	[7]
$FAMAPbBr_3$	CYTOP	N/A	N/A	0.02	N/A	N/A	[8]
$MAPbI_3$	CYTOP	N/A	N/A	1.8	N/A	N/A	[9]
$MAPbI_3$	Al_2O_3	<−2	N/A	23.2	N/A	$\sim10^4$	[10]

Table S3. EPMA result of the 2D/3D core–shell thin film (2D:3D=1:6) corresponding to the sample shown in Fig. 4e.

	Sn (at%)	I (at%)	Sn/I
Bright region (grain)	19.94	53.82	0.37
Dark region (boundary)	13.89	44.11	0.31

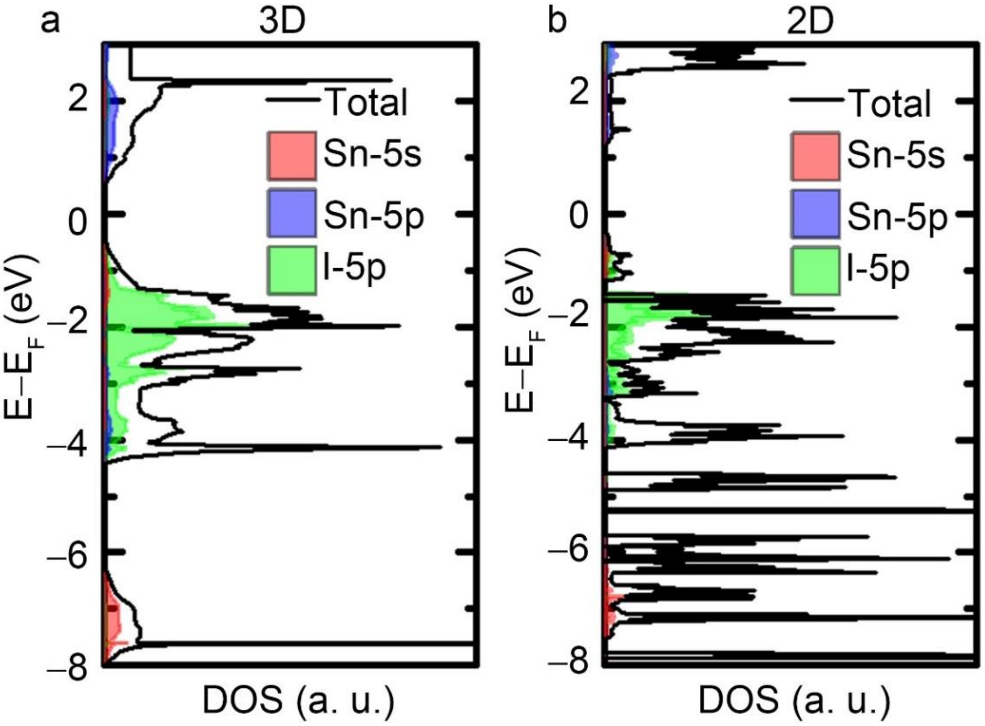

Figure S1. Density of states (DOS): (a) DOS profiles of 3D $FASnI_3$ and (b) 2D PEA_2SnI_4.

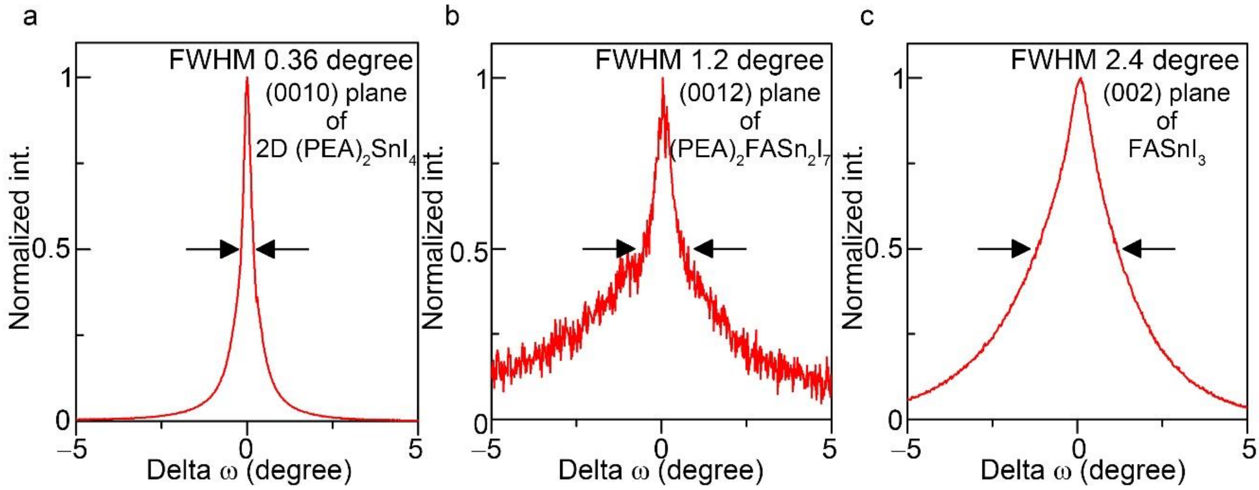

Figure S2. X-ray rocking curves of the (a) PEA_2SnI_4 thin film and (b and c) 2D/3D thin film (2D:3D = 1:6); (a) (0010) plane of PEA_2SnI_4, (b) (0012) plane of $PEA_2FASn_2I_7$, and (c) (002) plane of $FASnI_3$.

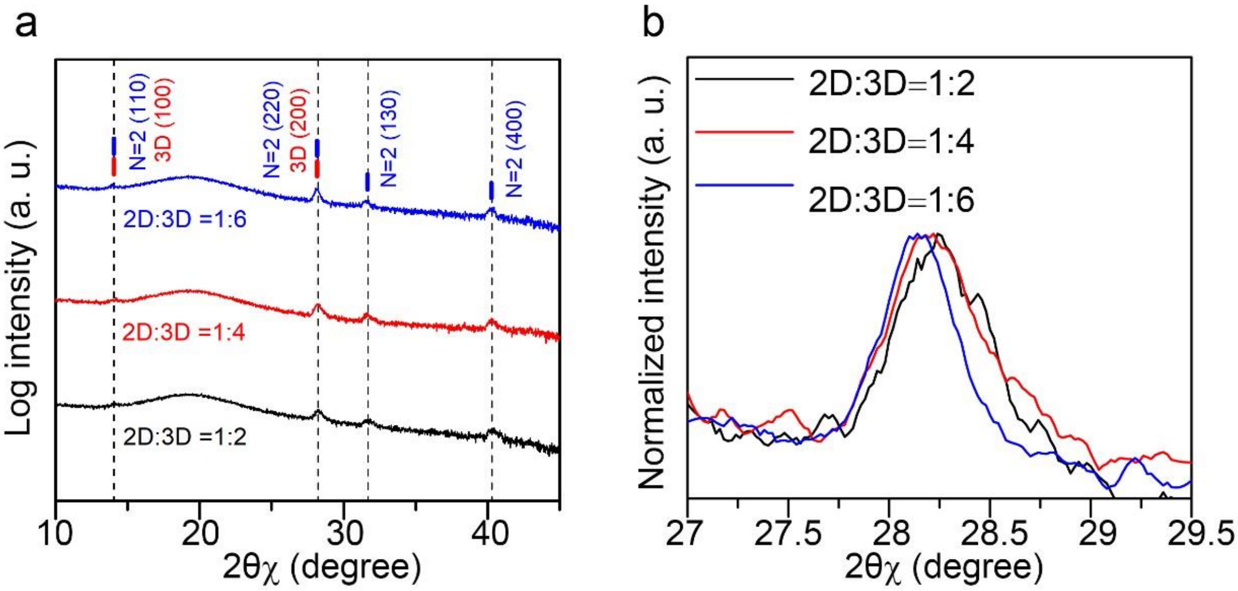

Figure S3. In-plane XRD with different 2D/3D mixing ratios. (a) In-plane XRD patterns; (b) magnified quasi-2D (N=2) (220) and 3D (200) peaks.

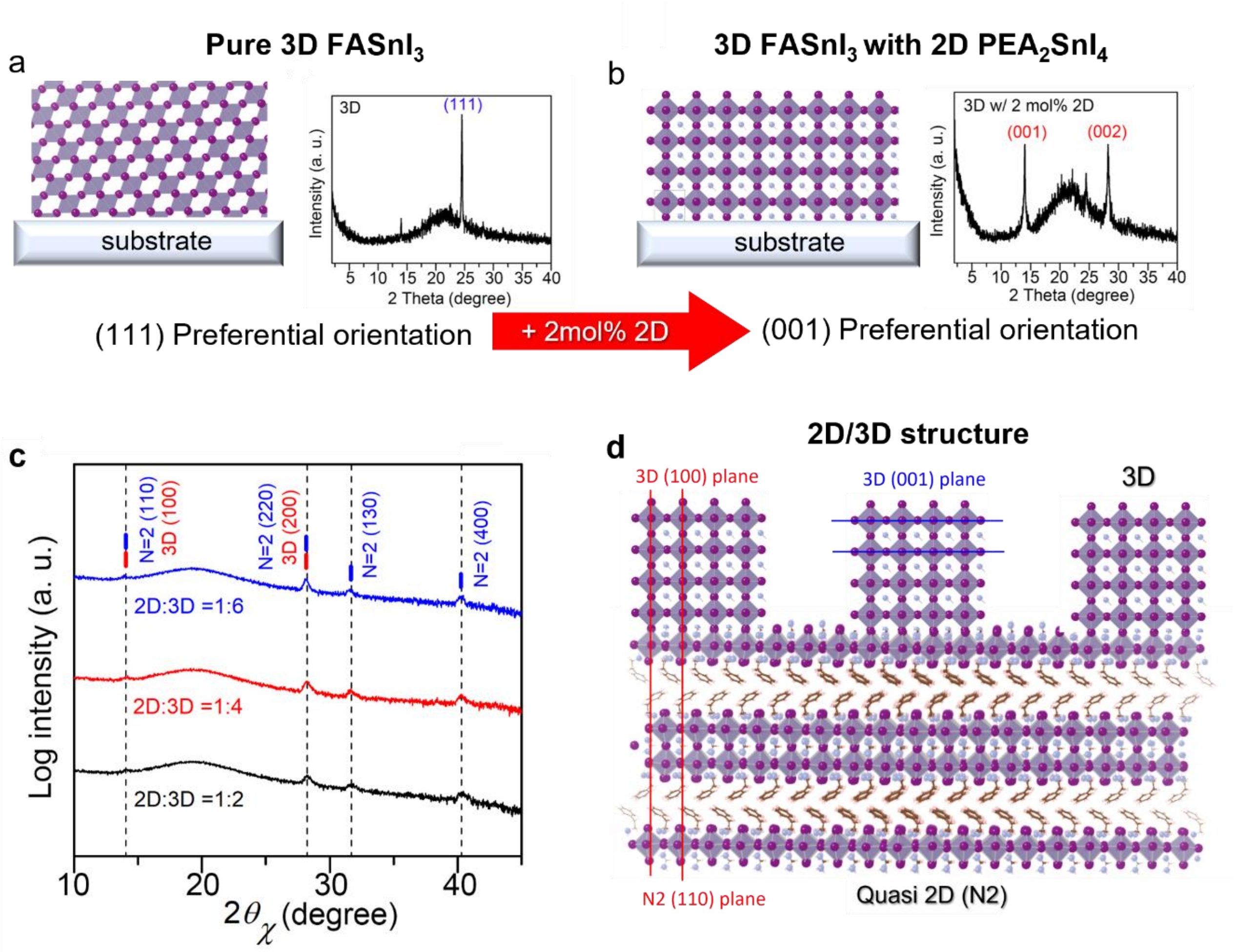

Figure S4. Comparison of the preferential orientations of 3D $FASnI_3$ thin films w/ and w/o 2D PEA_2SnI_4 and the expected 2D/3D structure. Out-of-plane XRD pattern of (a) pure 3D $FASnI_3$ and (b) 3D $FASnI_3$ with 2 mol% PEA_2SnI_4 (2D:3D mixing ratio = 1:50); (c) in-plane XRD pattern of 2D/3D thin films; (d) possible 2D/3D structure expected from the experimental results of a-c.

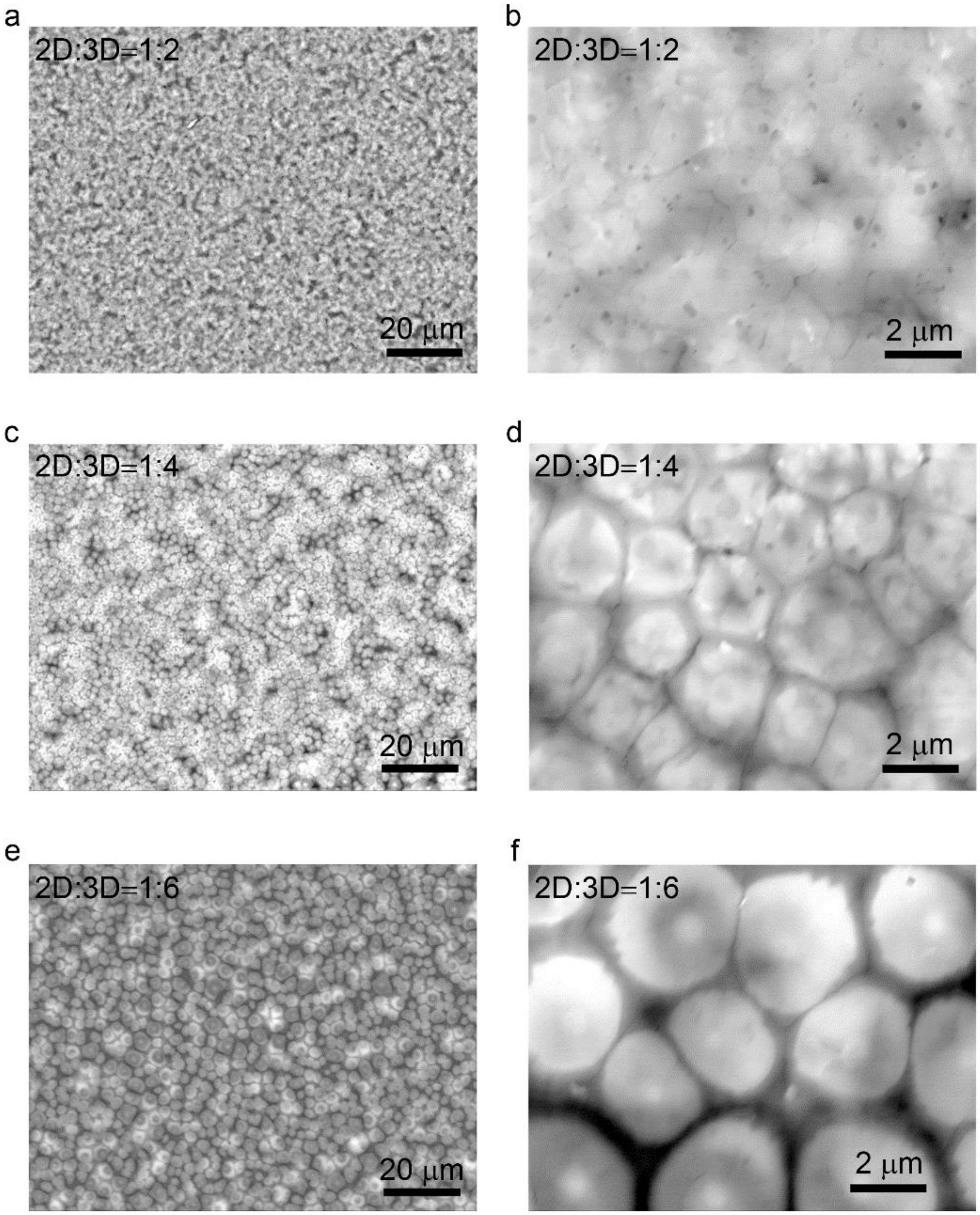

Figure S5. SEM images of the thin films with 2D:3D mixing ratios of (a), (b) 1:2, (c), (d) 1:4, and (e), (f) 1:6 in different scales.

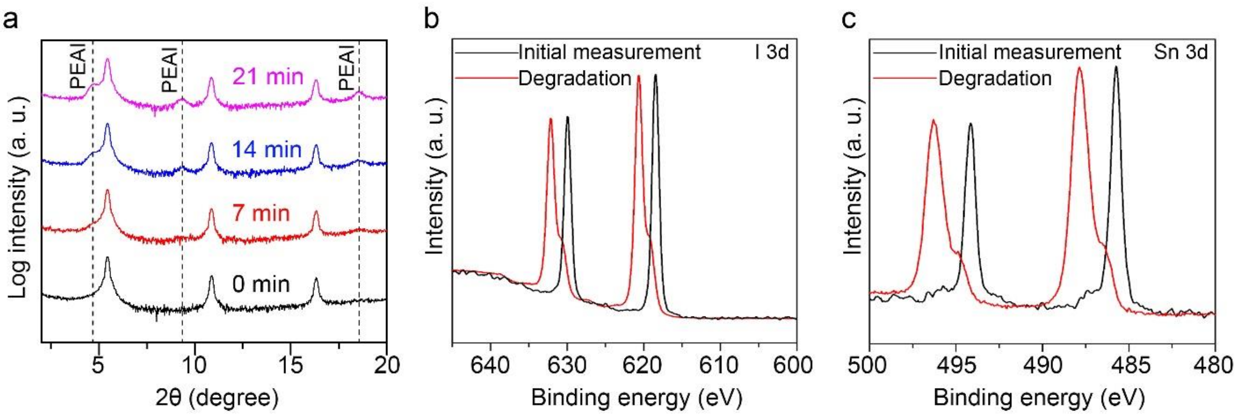

Figure S6. Time-dependent XRD and XPS results for PEA_2SnI_4 thin films. (a) XRD patterns. XPS spectra of the (b) I 3d and (c) Sn 3d peaks.

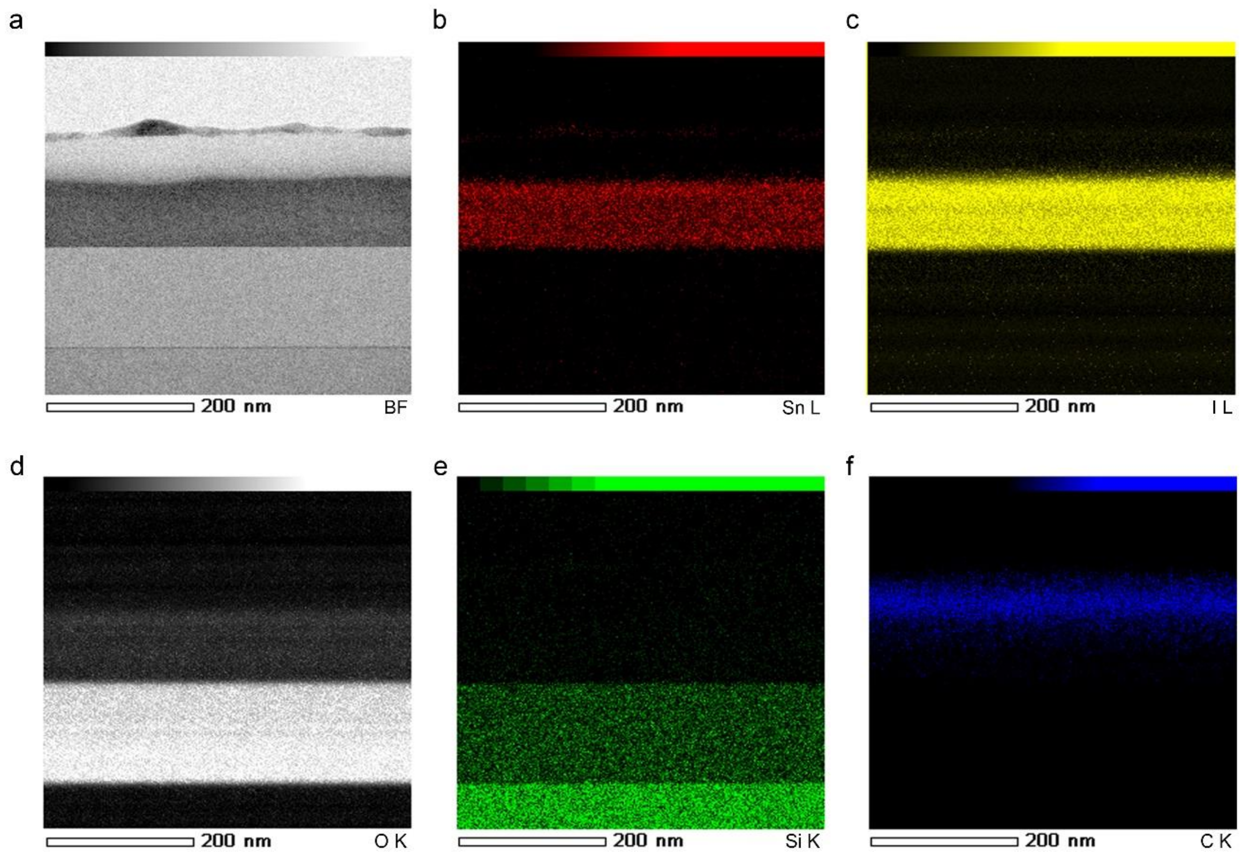

Figure S7. Cross-sectional TEM image and chemical composition of PEA_2SnI_4 passivated by 100 nm thick NPD and 100 nm Ag. (a) High-angle annular dark-field scanning transmission electron microscopy (HAADF-STEM) image. (b-f) Energy-dispersive X-ray spectroscopy (EDS) mapping.

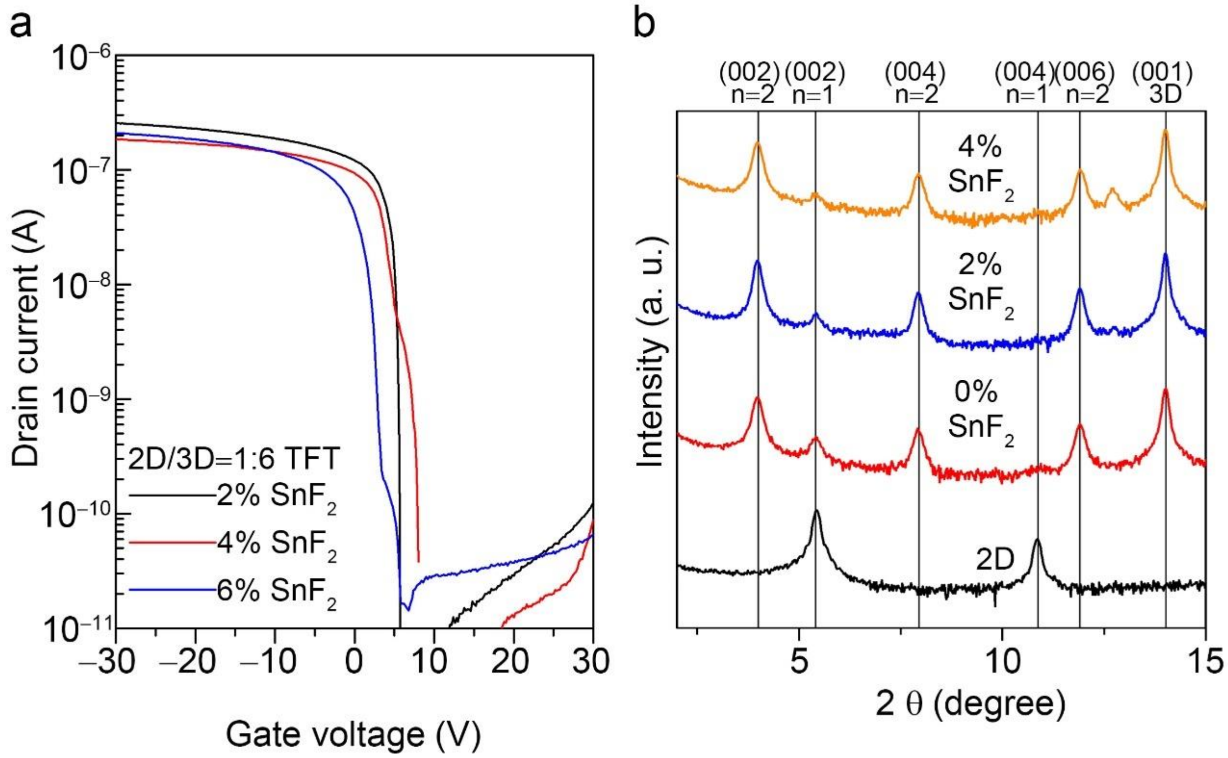

Figure S8. TFT performances and crystallinity variations of 2D:3D =1:6 with different amounts of SnF_2 additives. (a) Transfer curves of 2D/3D TFT (2D:3D =1:6) with different SnF_2 contents; (b) XRD results of the 2D/3D thin films (2D:3D =1:6) with different SnF_2 contents.

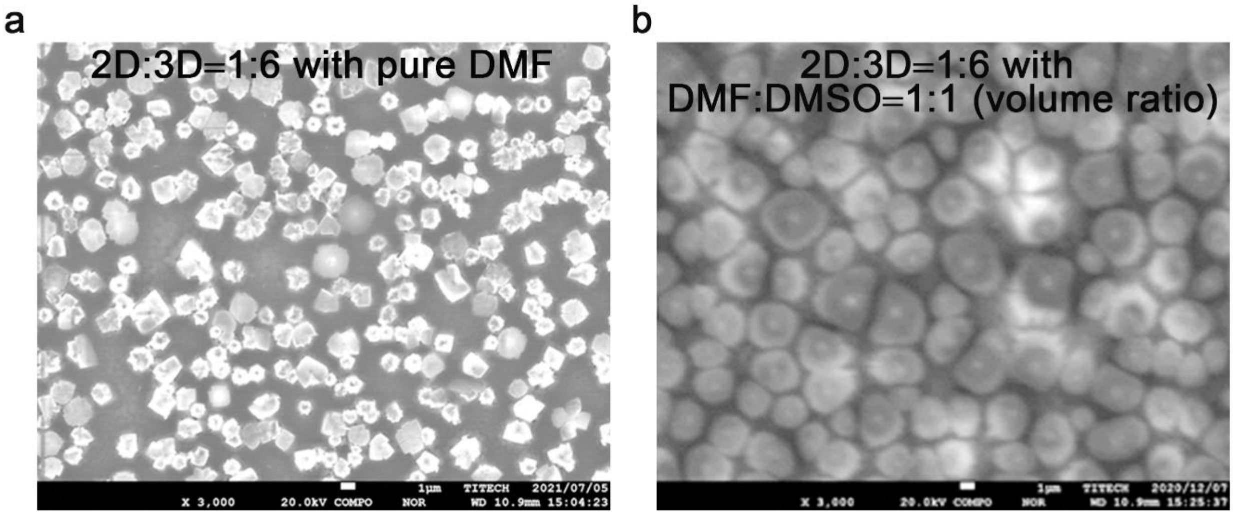

Figure S9. Morphologies of 2D:3D =1:6 dissolved in different solvents: (a) DMF and (b) DMF:DMSO = 1:1 (volume ratio).

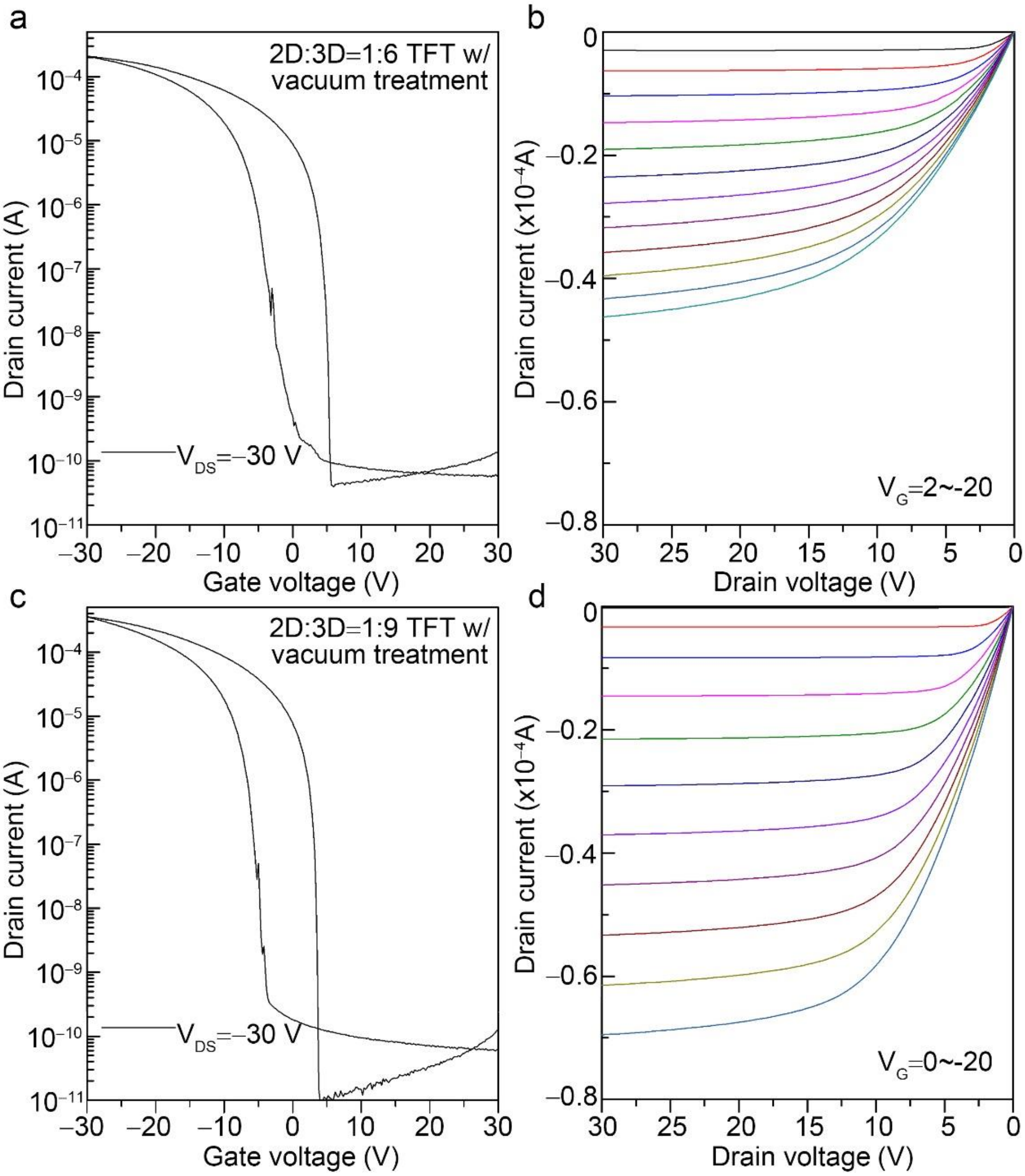

Figure S10. Hysteresis of the 2D/3D core–shell TFTs: Transfer curve: (a) 2D:3D = 1:6, (c) 2D:3D = 1:9. Output curve: (b) 2D:3D = 1:6, (d) 2D:3D = 1:9.

References

[1] H. Zhu, A. Liu, K. I. Shim, J. Hong, J. W. Han, Y. Y. Noh, *Adv. Mater.* **2020**, *32*, 2002717.

[2] C. R. Kagan, D. B. Mitzi, C. D. Dimitrakopoulos, *Science* **1999**, *286*, 945.

[3] T. Matsushima, S. Hwang, A. S. D. Sandanayaka, C. Qin, S. Terakawa, T. Fujihara, M. Yahiro, C. Adachi, *Adv. Mater.* **2016**, *28*, 10275.

[4] Y. Gao, Z. Wei, P. Yoo, E. Shi, M. Zeller, C. Zhu, P. Liao, L. Dou, *J. Am. Chem. Soc.* **2019**, *141*, 15577.

[5] H. Zhu, A. Liu, H. L. Luque, H. Sun, D. Ji, Y. Y. Noh, *ACS Nano* **2019**, *13*, 3971.

[6] Y. Reo, H. Zhu, J. Y. Go, K. I. Shim, A. Liu, T. Zou, H. Jung, H. Kim, J. Hong, J. W. Han, Y. Y. Noh, *Chem. Mater.* **2021**, *33*, 2498.

[7] S. Shao, W. Talsma, M. Pitaro, J. Dong, S. Kahmann, A. J. Rommens, G. Portable, M. A. Loi, *Adv. Funct. Mater.* **2021**. *31*, 2008478.

[8] S. P. Senanayak, M. A. Jalebi, V. S. Kamboj, R. Carey, R. Shivanna, T. Tian, G. Schweicher, J. Wang, N. Giesbrecht, D. D. Nuzzo, H. E. Beere, P. Docampo, D. A. Ritchie, D. F. Jimenez, R. H. friend, H. Sirringhaus, *Sci. Adv.* **2020**, 6.

[9] X. J. She, C. Chen, G. Divitini, B. Zhao, Y. Li, J. Wang, J. F. Orri, L. Cui, W. Xu, J. Peng, S. wang, A. Sadhanala, H. Sirringhaus, *Nat. Electron.* **2020**, *3*, 694.

[10] S. Jana, E. Carlos, S. Panigrahi, R. Martins, E. Fortunato, *ACS Nano* **2020**, *14*, 14790.
